# Using Spherical-Harmonics Expansions for Optics Surface Reconstruction from Gradients

**DOI:** 10.3390/s17122780

**Published:** 2017-11-30

**Authors:** Juan Manuel Solano-Altamirano, Alejandro Vázquez-Otero, Danila Khikhlukha, Raquel Dormido, Natividad Duro

**Affiliations:** 1 Facultad de Ciencias Químicas, Benemérita Universidad Autónoma de Puebla, 14 Sur y Av. San Claudio, Col. San Manuel, Puebla 72520, Mexico; 2 MSD IT Global Innovation Center s.r.o., Svornosti 3321/2, 150 00 Prague 5, Czech Republic; 3 ELI Beamlines, Institute of Physics ASCR, Za Radnicí 835, 252 41 Dolní Břežany, Czech Republic; 4 Department of Computer Sciences and Automatic Control, UNED, C/Juan del Rosal, 16, 28040 Madrid, Spain; raquel@dia.uned.es (R.D.); nduro@dia.uned.es (N.D.)

**Keywords:** wavefront reconstruction from gradients, surface reconstruction from gradients, spherical harmonics, zernike-polynomials, algorithm

## Abstract

In this paper, we propose a new algorithm to reconstruct optics surfaces (aka wavefronts) from gradients, defined on a circular domain, by means of the Spherical Harmonics. The experimental results indicate that this algorithm renders the same accuracy, compared to the reconstruction based on classical Zernike polynomials, using a smaller number of polynomial terms, which potentially speeds up the wavefront reconstruction. Additionally, we provide an open-source C++ library, released under the terms of the GNU General Public License version 2 (GPLv2), wherein several polynomial sets are coded. Therefore, this library constitutes a robust software alternative for wavefront reconstruction in a high energy laser field, optical surface reconstruction, and, more generally, in surface reconstruction from gradients. The library is a candidate for being integrated in control systems for optical devices, or similarly to be used in ad hoc simulations. Moreover, it has been developed with flexibility in mind, and, as such, the implementation includes the following features: (i) a mock-up generator of various incident wavefronts, intended to simulate the wavefronts commonly encountered in the field of high-energy lasers production; (ii) runtime selection of the library in charge of performing the algebraic computations; (iii) a profiling mechanism to measure and compare the performance of different steps of the algorithms and/or third-party linear algebra libraries. Finally, the library can be easily extended to include additional dependencies, such as porting the algebraic operations to specific architectures, in order to exploit hardware acceleration features.

## 1. Introduction

One of the main motivations to improve surface reconstruction techniques is to enhance wavefront sensors capabilities, which are instruments used to measure aberrations of incident wavefronts. In high-energy lasers production, these aberrations, i.e., imperfections, reduce the wavefront quality, and may also add undesirable effects to the produced beams, which, in turn, may affect the quality of the experimental outcomes wherein the laser is used. Furthermore, a wide range of technical fields benefit from accurate and efficient surface reconstruction algorithms, for instance the astronomical community, pioneering in developing wavefront reconstruction techniques for telescopes [1], measurements of eye aberrations [2], optical devices manufacturers, wherein the reconstruction plays a key role by identifying lens manufacturing errors, adaptive optics (AO) such as in microscopy [3] or data communication through the Earth’s atmosphere [4], lateral shearing interferometry [5,6,7], shape from shading [8], high energy laser (HEL) beam production control [9], or ophthalmology in refractive surgery [10].

In high-energy lasers production, opticians strongly prefer the Zernike polynomial set to reconstruct wavefronts and to decompose imperfections into well-known aberration components [11]. However, other sets have been proposed to be used for reconstructing surfaces (see, for instance, [12]). On the bright side, the Zernike-based reconstruction has been shown to outperform the iterative Fourier when reconstructing wavefront aberrations from slope data. However, on the other hand, noncircular pupils has posed a challenge to Zernike-based reconstruction, as the performance of this set for this pupils is questionable [13]. Furthermore, although Zernike polynomials constitute a complete set, hence any wavefront aberration can be decomposed in terms of them, and advantages of orthogonality are lost in noncircular cases. Moreover, the coefficients lose their physical meaning since the circle polynomials do not represent balanced aberrations for the noncircular pupil. In the library distributed along with this work, we provide a simple reconstruction algorithm based on the Legendre polynomial set for reconstructing surfaces in squared domains.

In addition, in producing high-energy lasers, the adaptive optics loop is critical, as in this process the quality of the wavefront is determined. The basic goal of the adaptive optics is easily stated: to measure the aberrations of an incoming wavefront and then cancel these out by applying compensating aberrations, all in real time. Therefore, it is desirable for the reconstruction algorithm to be as fast and accurate as possible. Unfortunately, high accuracy usually implies higher computational cost. The algorithm that we propose in this work, using Spherical Harmonics, may offer an alternative to the classical Zernike-based reconstruction algorithms, especially for automated processes wherein human intervention and analysis are not needed, but speed is, e.g., in adaptive optics [14].

The paper is organized as follows. In Section 2, we roughly describe how a Shack–Hartmann wavefront sensor works. In Section 3, we provide an overview of the modal reconstruction algorithm, and the generalities of our implemented version in OpenWavefrontReconstructor. The mathematical details of our new algorithm, using the Spherical Harmonics for circular domains, to reconstruct surfaces from gradients are given in Section 4. We also provide a few implementation details relative to the reconstruction algorithm that use classical Zernike polynomials for circular domains, and Legendre for square domains, in Section 5 and Section 6. In Section 7, we describe some general features of OpenWavefrontReconstructor. Numerical results and discussion are given in Section 8 and finally we close with conclusions and future work in Section 9.

## 2. Shack–Hartmann Wavefront Sensor

We will focus on improving the algorithms used internally by Shack–Hartmann wavefront sensors (the reader is referred to [2] and references therein for technical details on early designs). Roughly speaking, a wavefront sensor consists of three main parts: (a) an opto-mechanical device intended to provide measurements of the original light wavefront to be reconstructed; (b) its associated processing electronics; and (c) the software responsible for the wavefront reconstruction.

**Figure 1:**
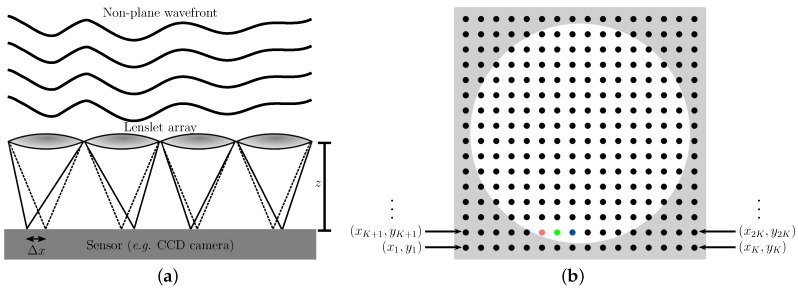
(**a**) Side view of a simplified Shack–Hartmann wavefront sensor scheme; (**b**) top view of the spotfield produced by a perfectly plane wavefront hitting a lenslet array.

The optomechanical part of a Shack–Hartmann wavefront sensor consists of a lenslet array and a light-sensing device (usually a charge-coupled device (CCD) or complementary metal-oxide-semiconductor (CMOS) camera). When a light wavefront hits the lenslet array (see Figure 1a), it generates a grid of small light spots (aka the spotfield, see Figure 1b) that are recorded by the light-sensing device. Through the intensity and shape of each light spot, the position of each center of mass can be found. In addition, if the wavefront is not plane, the produced spots deviate from the vertical position a distance Δx (see Figure 1a). Therefore, the wavefront slopes, dw/dx and dw/dy, can be evaluated through the distance between the lenslets and the sensor device, *z* (see Figure 1a), and Δx. These slopes constitute the information used to determine the shape of the wavefront through a reconstruction algorithm [2].

In this work, we will be focused on contibuting to the development of such wavefront reconstruction algorithms. However, the methods can also be used to reconstruct any surface whose slopes (or gradients) are known at a set of points. For this, one simply replaces the focal spots’ coordinates by the coordinates where the slopes are measured.

The spotfield produced by a wavefront sensor is commonly trimmed to fit a circular shape, such as the white zone depicted in Figure 1a. This is so because, in optics, most of the lenses used to focus light are circular; hence, the area illuminated by a focused light beam is circular as well. Nonetheless, the sensing device usually can record information in square shapes, such as the complete spotfield shown in Figure 1a, and even on rectangular shapes. Furthermore, noncircular shapes occur frequently; for instance, the cross section of a laser beam in high-power laser facilities is often square [15].

Since there exists a relation between the shape of the illuminated area and the domain whereupon polynomials sets are defined, in this paper, we will generically denote all shapes with the symbol *S*, and we will refer to both shape and actual domain indistinctively as domains. These domains are relevant to us because any set of orthogonal polynomials is complete only on a given domain.

## 3. Wavefront Reconstruction Method

There exist serveral methods to reconstruct a wavefront (or any surface) from slopes. In this paper, we will use the modal reconstruction method, as this is commonly used in Shack–Hartmann wavefront sensors. The modal reconstruction method is built upon the idea that a wavefront inciding on a sensing surface, *S*, can be recovered from slopes sampled at a set of points.

Furthermore, it is assumed that the wavefront, here denoted by w(x,y), can be decomposed into a polynomial series truncated to the term *J*:

(1) $$w\left(x,y\right)=\sum_{\alpha =1}^{J}A_{\alpha }\Xi_{\alpha }\left(x,y\right).$$

Here, $\left\{\Xi_{\alpha }\left(x,y\right)\right\}$ denotes a generalized set of functions (which is usually a set of polynomials orthogonal on the domain *S*), $A_{\alpha }$ the coefficients of the expansion, and α an ordering index that depends on the specific set $\left\{\Xi_{\alpha }\left(x,y\right)\right\}$. Traditionally, if *S* is a square, then

(2) $$\Xi_{\alpha }\left(x,y\right)=P_{k}\left(x\right)P_{l}\left(y\right),$$

where $P_{l}\left(t\right)$ are the Legendre Polynomials, and α=α(k,l) is some ordering function of the indices *k* and *l*. In the same fashion, if *S* is a circle, then

(3) $$\Xi_{\alpha }\left(x,y\right)=Z_{l,m}\left(r,\theta \right).$$

Here, $Z_{l,m}$ are the classical Zernike polynomials [11], whose radial degree is *l*, and α=α(l,m) is the respective ordering function of the indices *l* and *m* (see [16]). The specific form of α depends specifically on the type of polynomials used to expand the wavefront, and there is no universal convention (see Section 4, Section 5 and Section 6 for our specific implementation).

If Equation (1) is valid for a wavefront, w(x,y), its gradient at any point (x,y) is given by:

(4) $$\nabla w\left(x,y\right)=\sum_{\alpha }A_{\alpha }\left(\frac{\partial \Xi_{\alpha }\left(x,y\right)}{\partial x}\hat{ı}+\frac{\partial \Xi_{\alpha }\left(x,y\right)}{\partial y}\hat{j}\right),$$

where $\hat{ı}$ and $\hat{j}$ are unit vectors in *x* and *y* directions, respectively. Therefore, if ∇w(x,y) is known at a set of points, then the coefficients of the polynomial expansion can be recovered (see below). These slopes are precisely the information measured by a wavefront sensor.

### 3.1. Matrix Assembly

From the spot coordinates, $\left(x_{i},y_{i}\right)$ (see Figure 1b), we can assemble an array to contain the slopes of the wavefront at all sampled points $\left(x_{i},y_{i}\right)$, as follows:

(5) $$G\equiv \left[\begin{array}{c} \frac{\partial w(x_{i},y_{i})}{\partial x}\\ \frac{\partial w(x_{i},y_{i})}{\partial y} \end{array}\right]=\left[\begin{array}{c} \sum_{\alpha }A_{\alpha }\frac{\partial \Xi_{\alpha }\left(x_{1},y_{1}\right)}{\partial x}\\ \vdots \\ \sum_{\alpha }A_{\alpha }\frac{\partial \Xi_{\alpha }\left(x_{I},y_{I}\right)}{\partial x}\\ \sum_{\alpha }A_{\alpha }\frac{\partial \Xi_{\alpha }\left(x_{1},y_{1}\right)}{\partial y}\\ \vdots \\ \sum_{\alpha }A_{\alpha }\frac{\partial \Xi_{\alpha }\left(x_{I},y_{I}\right)}{\partial y} \end{array}\right].$$

In Equation (5), the coefficients $A_{\alpha }$ can be fitted to match the experimental slopes, and *i* is an index related to each focal spot (see Figure 1b). In the rest of this section, we will assume that our expansion has *J* terms, i.e., as in Equation (1), and that there are *I* sampled points whose coordinates are $\left(x_{i},y_{i}\right)$, i=1,⋯,I.

Similarly as for the ordering index α, there is no universal convention regarding the order of the coordinates of the focal spots. For square domains, the order of the nodes may be given as indicated in Figure 1b (here the grid has I=K×K spotlights), and for circular domains, the numbering may be given as $\left(x_{1},y_{1}\right)$ [red point], $\left(x_{2},y_{2}\right)$ [green point], $\left(x_{3},y_{3}\right)$ [blue point], etc. Fortunately, reconstruction algorithms do not depend on the specific ordering system of the focal spots; however, they depend on how exactly the coordinates of each node are known.

Equation (5) can be rewritten as:

(6) G=MA.

Here, A is the array of the expansion coefficients $A_{\alpha }$:

(7) $$A=\left(\begin{array}{c} A_{1}\\ A_{2}\\ \vdots \\ A_{J} \end{array}\right),$$

and the matrix M (whose dimension is 2I×J) is constructed as:

(8) $$M=\left(\begin{array}{ccc} \frac{\partial \Xi_{1}\left(x_{1},y_{1}\right)}{\partial x} & \cdots & \frac{\partial \Xi_{J}\left(x_{1},y_{1}\right)}{\partial x}\\ \vdots & \ddots & \vdots \\ \frac{\partial \Xi_{1}\left(x_{I},y_{I}\right)}{\partial x} & \cdots & \frac{\partial \Xi_{J}\left(x_{I},y_{I}\right)}{\partial x}\\ \frac{\partial \Xi_{1}\left(x_{1},y_{1}\right)}{\partial y} & \cdots & \frac{\partial \Xi_{J}\left(x_{1},y_{1}\right)}{\partial y}\\ \vdots & \ddots & \vdots \\ \frac{\partial \Xi_{1}\left(x_{I},y_{I}\right)}{\partial y} & \cdots & \frac{\partial \Xi_{J}\left(x_{I},y_{I}\right)}{\partial y} \end{array}\right).$$

### 3.2. Least-Squares Method and Singular Value Decomposition

The coefficients $A_{\alpha }$ can be found by the least-squares method, applied to Equation (6). For this, in the C++ library distributed along with this paper, we use the Singular Value Decomposition theorem [17,18], which states that any m×n real matrix M can be decomposed as

(9) $$M={\mathrm{USV}}^{T},$$

where in U ($=[u_{1}\;\cdots \;u_{n}]$) is an n×n matrix that orthogonally diagonalizes $M^{T}M$, V is an m×m ortogonal matrix, and the non-zero diagonal elements of the m×n matrix S are the non-zero eigenvalues of $M^{T}M$ corresponding to the column vectors ($u_{i}$) of U. Therefore, the coefficients of the expansion are given by

(10) $$A=VS^{-1}U^{T}G,$$

and the wavefront at any point is thus given by Equation (1).

In version 1.0.0 of OpenWavefrontReconstructor, we provide an implementation that uses the armadillo library [19] for solving Equation (10). The user may re-implement some of the functions of the library, should he/she desire to use a different linear algebra library. Optionally, the user may implement another third-party linear algebra library of his/her choice.

### 3.3. Wavefront Retrieving

Once the coefficients of the expansion, $A_{\alpha }$, are known, the values of the wavefront (or surface) at the sampled points, $\left\{w\left(x_{i},y_{i}\right)\right\}$, can be recovered through the following matrix equation:

(11) $$W=\left(\begin{array}{c} w(x_{1},y_{1})\\ \vdots \\ w(x_{I},y_{I}) \end{array}\right)=\mathrm{RA}=\left(\begin{array}{ccc} Z_{1}\left(x_{1},y_{1}\right) & \cdots & Z_{J}\left(x_{1},y_{1}\right)\\ \vdots & \ddots & \vdots \\ Z_{1}\left(x_{I},y_{I}\right) & \cdots & Z_{J}\left(x_{I},y_{I}\right) \end{array}\right)\left(\begin{array}{c} A_{1}\\ \vdots \\ A_{J} \end{array}\right).$$

Here, if there are *I* sampled points, $\left(\xi_{i},\eta_{i}\right)$, the matrix R has dimensions I×J. Of course, the wavefront can also be obtained at any point through Equation (1).

A performance remark: once the matrices $VS^{-1}U^{T}$ and R are generated during the first reconstruction, subsequent reconstructions can be carried out with a much smaller computational cost, using the same matrices. This is valid only if the focal spots’ coordinates do not change from reconstruction to reconstruction, which is true for wavefront sensors. For instance, the coefficients of a new wavefront expansion, $A^{\prime }$, whose slopes are given by a new vector $G^{\prime }$, are computed as $A^{\prime }=VS^{-1}U^{T}G^{\prime }$, and therefore the respective wavefront values are given by $W^{\prime }=RA^{\prime }$.

## 4. Half Circular Harmonics

**Figure 2:**
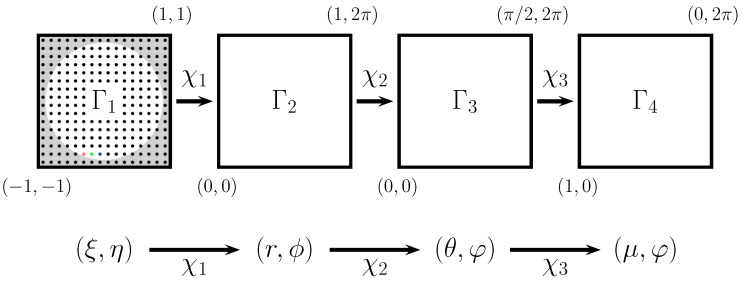
The mappings to transform the coordinates from the original Cartesian set $\Gamma_{1}\;=\;\left\{\left(\xi ,\eta \right)|\;\xi^{2}+\eta^{2}<1\right\}$ to the target set $\Gamma_{3}=\left\{(\mu ,\varphi )|1>\mu >0\;\;\&\;\;0<\varphi <2\pi \right\}$.

In this section, we describe the design and implementation of the new algorithm for reconstructing wavefronts using Spherical Harmonics as the orthogonal polynomial set mapped onto a circle. For this, we apply four consecutive mappings $\chi_{0}$, $\chi_{1}$, $\chi_{2}$, and $\chi_{3}$, which transforms the original coordinates until the coordinates are suitable for using half of the domain of the Spherical Harmonics (see Figure 2). The complete mapping from (x,y) to (μ,φ) is given by $\chi_{3}\circ \chi_{2}\circ \chi_{1}\circ \chi_{0}$, and we will refer to this mapped basis set as Half Circular Harmonics.

To use the library, the user must provide its own implementation of $\chi_{0}$, i.e., the library performs the map $\chi_{3}\circ \chi_{2}\circ \chi_{1}$, and all their related transformations. The coordinates of the grid must be normalized, i.e., the spot centers need to have 0<r<1 (which implies ξ∈[−1,1], η∈[−1,1], and $(\xi^{2}+\eta^{2})<1$).

The simple direct and indirect maps $\chi_{i}$ are given by:

(12) $$\begin{array}{ccc} \chi_{0}=\left\{\begin{array}{cc} \xi = & \frac{2x-x_{M}-x_{m}}{x_{M}-x_{m}}\\ \eta = & \frac{2y-y_{M}-y_{m}}{y_{M}-y_{m}} \end{array},\right. & \; & \chi_{0}^{-1}=\left\{\begin{array}{cc} x= & \frac{x_{M}-x_{m}}{2}\xi +\frac{x_{M}+x_{m}}{2}\\ y= & \frac{y_{M}-y_{m}}{2}\eta +\frac{y_{M}+y_{m}}{2} \end{array},\right. \end{array}$$

(13) $$\begin{array}{ccc} \chi_{1}=\left\{\begin{array}{cc} r= & \sqrt{\xi^{2}+\eta^{2}}\\ \phi = & \mathrm{atan}(\eta /\xi ) \end{array},\right. & \; & \chi_{1}^{-1}=\left\{\begin{array}{cc} \xi = & r\cos\phi \\ \eta = & r\sin\phi \end{array},\right. \end{array}$$

(14) $$\begin{array}{ccc} \chi_{2}=\left\{\begin{array}{cc} \theta = & \frac{\pi }{2}r\\ \varphi = & \phi \end{array},\right. & \; & \chi_{2}^{-1}=\left\{\begin{array}{cc} r= & \frac{2}{\pi }\theta \\ \phi = & \varphi \end{array},\right. \end{array}$$

(15) $$\begin{array}{ccc} \chi_{3}=\left\{\begin{array}{cc} \mu = & \cos\theta \\ \varphi = & \phi \end{array},\right. & \; & \chi_{3}^{-1}=\left\{\begin{array}{cc} \theta = & \arccos\mu \\ \phi = & \varphi \end{array},\right. \end{array}$$

thus the composite direct and indirect maps $\chi_{3}\circ \chi_{2}\circ \chi_{1}$ are

(16) $$\begin{array}{ccc} \chi_{3}\circ \chi_{2}\circ \chi_{1} & = & \left\{\begin{array}{cc} \mu = & \cos\left(\frac{\pi }{2}\sqrt{\xi^{2}+\eta^{2}}\right),\\ \varphi = & \mathrm{atan}\left(\eta /\xi \right), \end{array}\right. \end{array}$$

(17) $$\begin{array}{ccc} \chi_{1}^{-1}\circ \chi_{2}^{-1}\circ \chi_{3}^{-1} & = & \left\{\begin{array}{cc} \xi = & \frac{2}{\pi }\arccos\mu \cos\varphi =\frac{2}{\pi }\theta \cos\varphi ,\\ \eta = & \frac{2}{\pi }\arccos\mu \sin\varphi =\frac{2}{\pi }\theta \sin\varphi . \end{array}\right. \end{array}$$

For our purposes, we will also need the chain rule derivatives relative to the maps $\chi_{0}$ and $\chi_{3}\circ \chi_{2}\circ \chi_{1}$, which are given by:

(18) $$\begin{array}{ccc} \frac{\partial }{\partial \xi } & = & \frac{x_{M}-x_{m}}{2}\frac{\partial }{\partial x},\\ \frac{\partial }{\partial \eta } & = & \frac{y_{M}-y_{m}}{2}\frac{\partial }{\partial y}, \end{array}$$

(19) $$\begin{array}{ccc} \frac{\partial }{\partial \xi } & = & -\frac{\pi \sqrt{1-\mu^{2}}}{2}\cos\varphi \frac{\partial }{\partial \mu }-\frac{\pi \sin\varphi }{2\arccos\mu }\frac{\partial }{\partial \varphi },\\ \frac{\partial }{\partial \eta } & = & -\frac{\pi \sqrt{1-\mu^{2}}}{2}\sin\varphi \frac{\partial }{\partial \mu }+\frac{\pi \cos\varphi }{2\arccos\mu }\frac{\partial }{\partial \varphi }. \end{array}$$

Since OpenWavefrontReconstructor uses the circular domain $\Gamma_{1}$, the user must re-scale the gradients using the chain rule (18); here, we assume that the Cartesian coordinates inside $\Gamma_{1}$ are known. In the rest of the section, we describe the OpenWavefrontReconstructor’s internal procedure to reconstruct a wavefront (or a surface) defined on the domain $\Gamma_{1}$.

The sensor should provide (possibly after applying the map $\chi_{0}$) the gradients of the wavefront of a finite set of points contained in $\Gamma_{1}$, which must be ordered into an array of the type G (see Equation (5)). In the library, this ordering is performed by a function whose arguments are the following two array:

(20) $$G_{\xi }=\left[\frac{\partial w(\xi_{i},\eta_{i})}{\partial \xi }\right]=\left[\frac{x_{M}-x_{m}}{2}\frac{\partial w(x_{i},y_{i})}{\partial x}\right],$$

and

(21) $$G_{\eta }=\left[\frac{\partial w(\xi_{i},\eta_{i})}{\partial \eta }\right]=\left[\frac{y_{M}-y_{m}}{2}\frac{\partial w(x_{i},y_{i})}{\partial y}\right].$$

Here, the index *i* is the ordering index, and $x_{m}$, $x_{M}$, $y_{m}$, and $y_{M}$ are the minimum and maximum values of *x* and *y* (see Figure 1b).

Internally, OpenWavefrontReconstructor assembles the matrix M (see Equation (8)), applying the chain rules, Equation (19), and using the pairs $\left(\mu_{i}\left(\xi_{i},\eta_{i}\right),\varphi_{i}\left(\xi_{i},\eta_{i}\right)\right)$. Subsequently, it solves the equation system (10) for the coefficients of the expansion (see Equation (7)). Afterwards, the user can retrieve the coefficients, request the wavefront reconstruction, and retrieve the values of the wavefront at the coordinates ($\xi_{i},\eta_{i}$) (with which the matrix M was originally generated). For the latter, OpenWavefrontReconstructor internally applies the inverse map $\chi_{1}^{-1}\circ \chi_{2}^{-1}\circ \chi_{3}^{-1}$ to the coordinates and returns the wavefront, using Equation (1), in the original coordinates (ξ,η).

After the first reconstruction, the user may request additional reconstructions if the coordinates of the spots do not change (see Section 3.3). This procedure would considerably decrease the computation time if the reconstruction is performed in a loop, which is quite common in adaptive optics, and in high-energy lasers production (especially in the so-called adaptive optics loop, which is a coupled system of a wavefront sensor and a deformable mirror, whose purpose is to correct aberrations originated during the production of the laser beams). After the first reconstruction (which requires assembling the matrix M, the singular value decompositon shown in Equation (9), and generating the matrix R defined in Equation (11)), the computational cost of subsequent reconstructions reduces to perform a matrix-vector product (i.e., the product RA given in Equation (11)).

Each term of the matrix ($R_{\alpha ,i}$) is of the form:

(22) $$R_{\alpha ,i}\equiv Y_{\alpha }\left(\xi_{i},\eta_{i}\right)=Y_{n}^{m}\left(\mu_{i}\left(\xi_{i},\eta_{i}\right),\varphi_{i}\left(\xi_{i},\eta_{i}\right)\right).$$

In Equation (22), *n* is the principal order of the Spherical Harmonics, and the ordering index α=α(n,m) reproduces the following order: α(0,0)=1, α(1,−1)=2, α(1,0)=3, α(1,1)=4, α(2,−2)=5, α(2,−1)=6,… OpenWavefrontReconstructor uses this convention. In addition, for the internal numerical computations of OpenWavefrontReconstructor, we implemented the Real Spherical Harmonics (or Tesseral Spherical Harmonics), and we use the following normalization convention:

(23) $$Y_{n}^{m}\left(\mu ,\varphi \right)=\left\{\begin{array}{ccc} \frac{1}{\sqrt{\pi }}P_{n}^{\left|m\right|}\left(\mu \right)\sin\left(|m|\varphi \right), & \; & m<0,\\ \frac{1}{\sqrt{2\pi }}P_{n}^{0}\left(\mu \right), & \; & m=0,\\ \frac{1}{\sqrt{\pi }}P_{n}^{m}\left(\mu \right)\cos\left(m\varphi \right), & \; & m>0. \end{array}\right.$$

Therefore,

(24) $$\int_{-1}^{1}d\mu \int_{0}^{2\pi }Y_{n}^{m}\left(\mu ,\varphi \right)Y_{n^{\prime }}^{m^{\prime }}\left(\mu ,\varphi \right)d\varphi =\delta_{nn^{\prime }}\delta_{mm^{\prime }}.$$

## 5. Classical Zernike Decomposition

In the library, we implemented the classical Zernike polynomials to reconstruct wavefronts defined on circular domains [11]. We provide this in order both to use this reconstruction as a benchmark (i.e., to measure the quality of the reconstruction using Half Circular Harmonics below) and to provide the user with well-known reconstruction methods.

Briefly, the Zernike polynomials are defined as

(25) $$Z_{l}^{m}\left(\rho ,\phi \right)=\left\{\begin{array}{c} R_{l}^{m}\left(\rho \right)\cos\left(m\phi \right),\;l\mathrm{even},\\ R_{l}^{m}\left(\rho \right)\sin\left(m\phi \right),\;l\mathrm{odd}. \end{array}\right.$$

Here, *l* and *m* are nonnegative integers, l≥m, 0≤ρ≤1, 0≤ϕ≤2π, and $R_{l}^{m}\left(\rho \right)$ is given by

(26) $$R_{l}^{m}\left(\rho \right)=\sum_{k=0}^{(l-m)/2}\frac{{\left(-1\right)}^{k}\left(l-k\right)!}{k!((l+m)/2-k)!((l-m)/2-k)!}\rho^{l-2k}.$$

In Equation (26), it has been assumed, by definition, that l−m is even. If l−m is odd, then $R_{l}^{m}=0$, also by definition. For further details, the interested reader may consult, for instance, Ref. [11].

Our implementation uses the Noll’s sequential indices [16] for α, which reproduces the following sequence: α(0,0)=1, α(1,1)=2, α(1,−1)=3, α(2,2)=4, α(2,0)=5, α(2,−2)=6, α(3,3)=7, α(3,1)=8,… We will refer to the radial degree *l* (see Equations (3), (25) and (26)) as the principal order of the Zernike polynomials.

In version 1.0.0 of OpenWavefrontReconstructor, the user is responsible for renormalizing the focal spot coordinates, such that they are defined on circular domain whose radius is <1, which can be done using Equations (12) and (18).

Internally, OpenWavefrontReconstructor uses the same procedure as described in Section 4, but exchanging the Half Circular Harmonics by Zernike polynomials of the form shown in Equation (3).

## 6. Legendre Polynomials for Square and Rectangular Domains

To reconstruct wavefronts defined on square domains, in OpenWavefrontReconstructor, we implemented the classical Legendre polynomials as decribed by Equation (2).

The user of OpenWavefrontReconstructor must provide a custom implementation of the scaling transformations, using Equations (12) and (18). The rest of the reconstruction procedure is carried out automatically, and there is no required order for the focal spot coordinates (see Figure 1b).

Wavefronts defined on rectangular domains can also be reconstructed using the direct map $\chi_{0}$, i.e., Equations (12) and (18), which maps the rectangle into a unit square. The reconstruction would render the square wavefront, and the user would just use the inverse map $\chi_{0}^{-1}$, i.e., Equation (12), in order to recover the original coordinates. However, since the order in which the coordinates are arranged does not change during the reconstruction algorithms, the user does not need to recompute the original coordinates, but only associate the wavefront values through the index of the arrays (let us recall that the arrays are ordered, see Equations (20) and (21)).

Internally, OpenWavefrontReconstructor uses the same procedure as described in Section 4, but replacing the Half Circular Harmonics by products of Legendre polynomials of the form shown in Equation (2). The ordering index function, α(k,l), for this set reproduces the sequence α(0,0)=1,…, α(0,N)=N+1, α(1,0)=N+2,…

## 7. OpenWavefrontReconstructor Implementation Details

The source code of the herein described wavefront reconstruction algorithms has been made available for public download at [20] under the GPLv2 license. The library OpenWavefrontReconstructor is written in C++ with a high degree of configuration, and it is aimed to facilitate future research in the field, as well as to provide a *ready-to-go* framework for being used in control systems. The library contains the following capabilities:

1. A mock-up generator of various types of incident wavefronts (see Section 8.1 for specific functions).
2. Implementation of different wavefront reconstruction algorithms: Zernike polynomials and Half Circular Harmonics for circular domains, and Legendre polynomials for square domains.
3. Runtime selection of the linear algebra library to perform the algebraic computations
4. Time profiling of the linear algebraic operations (mainly the CPU time (*aka* the process time) needed to generate the matrices M, and R defined in Equations (8) and (11), and also to record the time of the matrix-vector product for performing the operation described in Equation (11)).

All software entities are fully decoupled, therefore OpenWavefrontReconstructor can be easily modified or extended. This also applies to the *class* in charge of performing the algebraic computations. Additionally, porting the linear algebra functions to specific hardware architectures is part of our future work, which is aimed to decrease the CPU times below the millisecond for the operations associated with Equations (10) and (11). According to our experience, this will make the OpenWavefrontReconstructor an interesting option in control systems where the reconstruction is required to be nearly real-time, such as in the adaptive optics [4,14].

**Figure 3:**
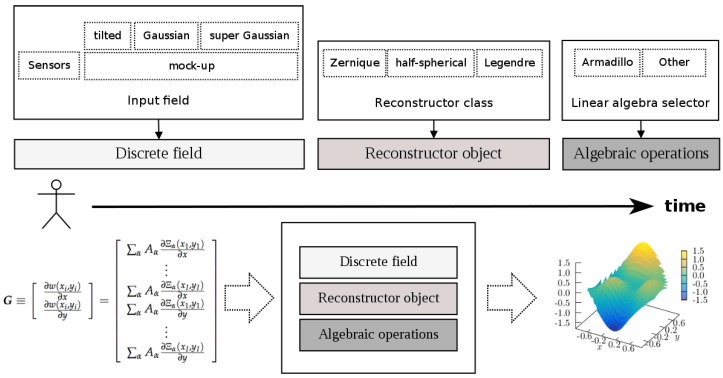
A visual representation of the different possibilities offered by the library for wavefront reconstruction.

The diagram depicted in Figure 3 provides the different options offered by the library, and it shows a typical work flow that can be described as follows:

1. The user can choose the input wavefront as a simulated optical field generated by the mock-up generator, or as a direct input from a sensor. Usually, the sensor vendors provide libraries that can be used to retrieve information such as the focal spots coordinates, wher the slopes are measured, and obviously these also provide the slopes. These coordinates and slopes are the inputs received by OpenWavefrontReconstructor, and are used to configure the matrices M and R defined by Equations (8) and (11).
2. Similarly, the user can choose the particular algorithm that will perform the wavefront reconstruction, which can be instantiated as an object from the available classes representing the different approaches (see the previous enumeration list—item 2—for the list of available polynomials).
3. Finally, the user can also select, at runtime, the linear algebra library to perform the linear algebraic operations. In version 1.0.0 of OpenWavefrontReconstructor, only the armadillo library is merged in the code; however, we plan to include more options, and the user can also implement the libraries of his/her preference. OpenWavefrontReconstructor’s design is intended to provide an easy-to-follow (nearly copy-paste) environment to implement new linear algebra libraries.

## 8. Numerical Results and Discussion

In this work, we present detailed numerical experiments exclusively for the Half Circular Harmonics, which are proposed by the authors. We compare the accuracy of the reconstruction against the results obtained with classical Zernike polynomials.

### 8.1. Testing Functions

**Figure 4:**
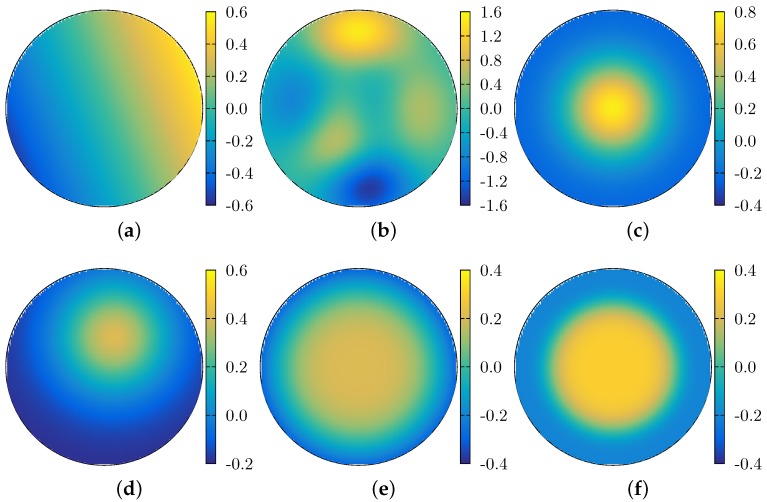
Topview of the wavefronts used to measure the reconstruction algorithm’s accuracy. The functions depicted are as follows. (**a**) a tilted plane, both around the *x*- and *y*-axis; (**b**) the function $f_{1}$ (see Equation (27)); (**c**) a centered Gaussian; (**d**) and off-centered Gaussian; (**e**) a centered super-Gaussian of order 4; and (**f**) a centered super-Gaussian of order 6.

For the tests and results presented in this section, we use the wavefronts depicted in Figure 4. Most of them were chosen because they are frequently encountered in the laser wavefront reconstruction problem. Figure 4a shows a tilted plane, both around the *x*- and *y*-axis. Figure 4b shows a combination of three terms, each of which is a polynomial times an off-centered Gaussian. We will refer to this function as the test $f_{1}$ function, and it is defined as follows:

(27) $$\begin{array}{ccc} f_{1}\left(x,y\right) & = & \frac{1}{5}\{3{\left(1-2x\right)}^{2}\exp\left(-4x^{2}-{\left(2y+1\right)}^{2}\right)-\frac{1}{3}\exp\left(-{\left(2x+1\right)}^{2}-{\left(2y\right)}^{2}\right) \end{array}$$

(28) $$\begin{array}{c} \;-10\left[\frac{2x}{5}-{\left(2x\right)}^{3}-{\left(2y\right)}^{5}\right]\exp\left(-{\left(2x\right)}^{2}-{\left(2y\right)}^{2}\right)\}. \end{array}$$

Figure 4c shows a centered Gaussian function, i.e., $w\left(x,y\right)=A\exp\left(-a(x^{2}+y^{2})\right)$, and Figure 4d shows an off-centered Gaussian, i.e., $w\left(x,y\right)=A\exp(-a[{\left(x-x_{0}\right)}^{2}+{\left(y-y_{0}\right)}^{2}])$. Figure 4e,f show Super Gaussian wavefronts of orders 4 and 6 (a super Gaussian of order *n* is defined as $w\left(x,y\right)=A\exp\left(-a(x^{n}+y^{n})\right)$, with *n* even).

### 8.2. Qualitative Reconstruction

**Figure 5:**
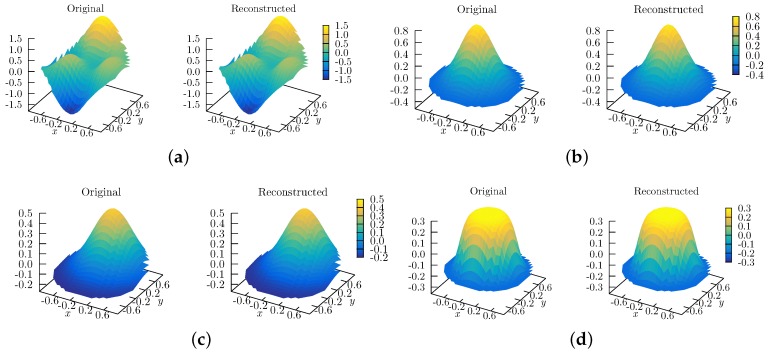
Qualitative reconstruction of several wavefronts, using Half Circular Harmonics, with J=81. The functions depicted are: (**a**) the test function $f_{1}$ (see Equation (27), and Figure 4b for the top view); (**b**) a centered Gaussian (see Figure 4c); (**c**) an off-centered Gaussian (see Figure 4d); and (**d**) a centered super-Gaussian of order 6 (see Figure 4f).

In Figure 5, we qualitatively compare the reconstruction of several wavefronts. The grids were generated using a wavefront simulator (i.e., the class MockWavefrontGenerator of OpenWavefrontReconstructor). The circular grid was obtained from an initial 30 × 30 square grid, from which only the points inside the unit circle are kept. This grid size corresponds to approximately the number of focal spots measured by a wavefront sensor. Neither the mock generator nor the reconstruction algorithms are exclusive to this size, but the user can setup any grid size.

We used J=81 polynomial terms to perform the reconstruction. This number was obtained by setting the maximum value, *N*, of the principal order of the Half Circular Harmonic to be N=8. The principal order can be easily identified by comparing it with the corresponding Spherical Harmonic, $Y_{n}^{m}\left(\mu ,\phi \right)$ (see also the text after Equation (22)).

### 8.3. Accuracy

**Figure 6:**
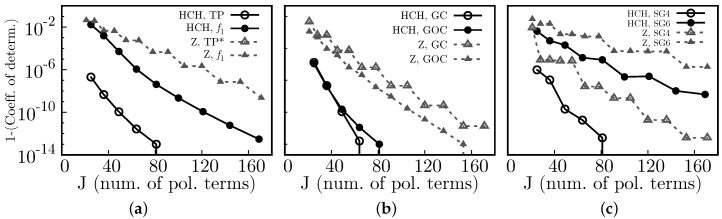
Compared accuracy (1-(Coefficient of determination)) vs. J (number of polynomial terms) between Half Circular Harmonics (HCH) and the Zernike (Z])polynomials, for the test functions (**a**) Tilted Plane (TP/TP*), $f_{1}$, (**b**) a centered Gaussian (GC), an off-centered Gaussian (GOC), and (**c**) super-Gaussians of order 4 (SG4) and 6 (SG6).

In Figure 6, we show the accuracy of the reconstruction algorithm using Half Circular Harmonics, via the coefficient of determination, *C*, which is defined as

(29) $$C\equiv 1-\frac{S_{\mathrm{res}}}{S_{\mathrm{tot}}}=1-\frac{\sum_{i}{\left(y_{i}-f_{i}\right)}^{2}}{\sum_{i}{\left(y_{i}-\bar{y}\right)}^{2}}.$$

Here, $S_{\mathrm{res}}$ is known as the sum of squares of residuals, and $S_{\mathrm{tot}}$ is the total sum of squares. In addition, in Equation (29), $\left\{y_{i}\right\}$ is the set of known values (i.e., our known test function), $\bar{y}$ the mean value of the set $\left\{y_{i}\right\}$, and $\left\{f_{i}\right\}$ the predicted (i.e., the reconstructed) values (see also [21]). Since the coefficients of determination are close to 1, we actually plot 1-*C* in semi-log scale. As a general trend, a smaller number of polynomial terms, *J*, is needed to obtain a given accuracy (compared to using Zernike polynomials). The curve for the Tilted Plane using Zernike polynomials, TP*, does not appear because for this case 1−C is close to the machine precision epsilon.

**Figure 7:**
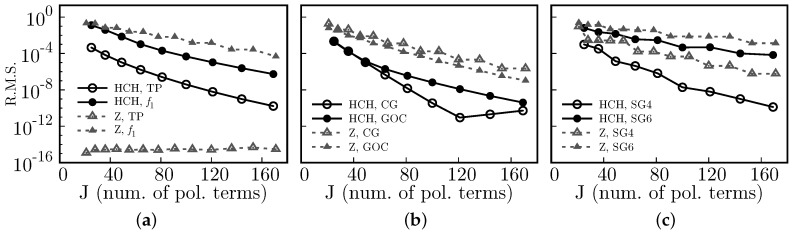
Compared Root Mean Square (R.M.S.) vs. J (number of polynomial terms) between Half Circular Harmonics (HCH) and the Zernike (Z) polynomials, for the test functions (**a**) Tilted Plane (TP), $f_{1}$, (**b**) a centered Gaussian (GC), an off-centered Gaussian (GOC), and (**c**) super-Gaussians of order 4 (SG4) and 6 (SG6).

In Figure 7, we show the R.M.S. for the same test set as in Figure 6. Here, we used the following definition:

(30) $${\left(R.M.S.\right)}^{2}=\frac{\sum_{i}{\left(y_{i}-f_{i}\right)}^{2}}{\sum_{i}y_{i}^{2}},$$

where $\left\{y_{i}\right\}$ and $\left\{f_{i}\right\}$ are the set of known and predicted values, respectively.

Except for the tilted plane, both accuracy measures indicate that using HCH increases the quality of the reconstructed surfaces. The tilted plane is perfectly described by Zernike because one of the Zernike polynomials is precisely a tilted plane.

### 8.4. Performance

Usually, matrix-vector products require O(2rs) floating point operations (here, *r* is the number of elements of the vector, and the matrix has dimensions r×s (see [17])). Therefore, reducing either the number of sampled focal spots, *I*, or the number of polynomias used for the expansion, *J*, may impact the total CPU time required to reconstruct wavefronts. Furthermore, if the coordinates of the focal spots (i.e., the coordinates where the slopes are sampled) do not change between consecutive reconstructions, then the computational cost should be considerably reduced. This can be seen from the following rationale. Performing singular value decompositions require $O(8IJ^{2}+8J^{3})$ flops, for a matrix of dimensions 2I×J [17]. On the other hand, the products $VS^{-1}U^{T}G$ (Equation (10)) and RA (Equation (11)) require O(4IJ) and O(2IJ). Hence, after performing the SVD, subsequent reconstructions require O(6IJ) flops.

**Figure 8:**
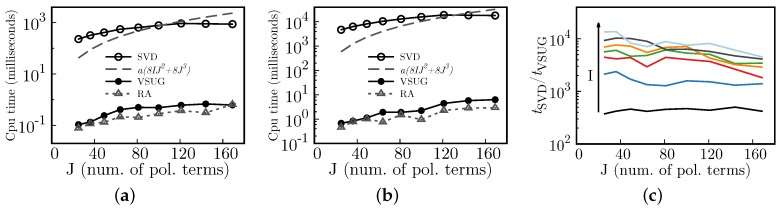
Processing times (CPU times) for different grid sizes (*I*), number of polynomial terms (*J*), and matrix operations. SVD is Singular Value Decomposition, VSUG represents the product $VS^{-1}U^{T}G$ (Equation (10)), and RA the product RA (Equation (11)). (**a**) matrix operations for a system with I=648; (**b**) matrix operations for a system with I=2724; (**c**) the ratio between SVD and VSGU CPU times, $t_{\mathrm{SVD}}/t_{\mathrm{VSGU}}$; the arrow indicates increasing *I*, and *I* takes the values I=276,648,1184,1876,2724,3720, and 4872. In (**a**,**c**), $a(8IJ^{2}+8J^{3})$ is the theoretical flops times a scaling constant *a*.

In Figure 8, we show the CPU times of different matrix operations for different *I* and *J*. The Singular Value Decomposition CPU times include the computation of the $M_{\alpha j}$ and $R_{\alpha j}$ terms (see Equations (8) and (11)). As expected, after the first reconstruction, further coefficient estimations (VSUG) and wavefront retrieving (RA) are carried out $∼10^{2}$ to $10^{4}$ faster (see Figure 6c), relative to the complete process (which takes approximately the combined $t_{\mathrm{SVD}}+t_{\mathrm{VSUG}}+t_{\mathrm{RA}}\approx t_{\mathrm{SVD}}$ CPU time). The ratio $t_{\mathrm{SVD}}/t_{\mathrm{RA}}$ increases as the system’s size, hence the performance improvement also increases with the system’s size.

## 9. Conclusions

We proposed a new algorithm to reconstruct wavefronts, and more generally surfaces, from gradients using the Spherical Harmonics mapped to work for unit circle domains, which we call Half Circular Harmonics. Relative to the use of classical Zernike polynomials, the same numerical accuracy can be obtained using ∼1/2 to 2/3 the number of polynomial terms. This might decrease the computational time spent to reconstruct wavefronts from slopes, which may be of interest to designers and manufacturers of wavefront sensors.

Additionally, we implemented the proposed reconstruction algorithm in a library (OpenWavefrontReconstructor) released to the public under the GPLv2 license terms. The library is designed to be used as a part of the wavefront sensors software, hence, in addition to the new polynomials (i.e., the Half Circular Harmonics), we also provide an implementation for reconstructing wavefronts, defined on a unit circle, using the classical Zernike polynomials. On the other hand, for square domains, we implemented the classical Legendre polynomials. Currently, OpenWavefrontReconstructor uses the linear algebra library armadillo to perform the matrix operations; however, its design is intended to provide an easy-to-follow (nearly copy-paste) environment to implement new linear algebra libraries. Futher additions to the library are planned as well, such as coupling other linear algebra libraries and additional polynomial sets for square domains. Finally, we also plan to port OpenWavefrontReconstructor to specific massively paralell hardware architectures, in order to decrease the reconstruction computing time.
